# Disparities in central line-associated bloodstream infection and catheter-associated urinary tract infection rates: An exploratory analysis

**DOI:** 10.1017/ice.2023.63

**Published:** 2023-11

**Authors:** Erin B. Gettler, Ibukunoluwa C. Kalu, Nwora L. Okeke, Sarah S. Lewis, Deverick J. Anderson, Becky A. Smith, Sonali D. Advani

**Affiliations:** 1 Division of Infectious Diseases, Department of Medicine, Duke University School of Medicine, Durham, North Carolina; 2 Duke Center for Antimicrobial Stewardship and Infection Prevention, Duke University Medical Center, Durham, North Carolina; 3 Division of Pediatric Infectious Diseases, Department of Pediatrics, Duke University School of Medicine, Durham, North Carolina; 4 Department of Population Health Sciences, Duke University School of Medicine, Durham, North Carolina

## Abstract

This retrospective review of 4-year surveillance data revealed a higher central line-associated bloodstream infection (CLABSI) rate in non-Hispanic Black patients and higher catheter-associated urinary tract infection (CAUTI) rates in Asian and non-Hispanic Black patients compared with White patients despite similar catheter utilization between the groups.

Health disparities are differences in the burden of disease, injuries, access, and opportunities that are related to economic, social, political, and environmental disadvantages.^1^ Inequities faced by minoritized racial and ethnic groups contribute to health disparities and poorer health outcomes across many domains. Exacerbation of disparities during the coronavirus disease 2019 (COVID-19) pandemic has helped raise awareness of health inequities.^2,3^ However, there is limited evidence related to disparities in healthcare-associated infections (HAI) based on age, hospital type, and race or ethnicity.^4–6^ Additionally, there are no national standards on whether race and ethnicity data should be included in HAI surveillance systems and how these data should be equitably used to guide prevention efforts. The objective of this study was to explore the differences in the rates of central line-associated bloodstream infection (CLABSI) and catheter-associated urinary tract infection (CAUTI) in a representative cohort of hospitalized patients at a large academic medical center.

## Methods

### Study design and setting

We performed a retrospective cohort study of prospectively collected CLABSI and CAUTI surveillance data in adult inpatients admitted to a 957-bed academic medical center in Durham, North Carolina, between January 1, 2018, and December 31, 2021. The study was determined to be exempt by the institutional review board of Duke University Health System with a waiver of consent (no. Pro00104110).

### Definitions

CLABSIs and CAUTIs were defined using Centers for Disease Control and Prevention’s National Health Safety Network (NHSN) surveillance criteria. These outcomes were analyzed by race and ethnicity as documented in the electronic medical record (EMR). All patients who either self-reported or were documented as Hispanic or Latinx ethnicity were considered as such regardless of race. For those reporting non-Hispanic ethnicity, patients were classified into 6 race categories: American Indian/Alaska Native, Asian, Black or African American, Native Hawaiian/Pacific Islander, Other, and White. The Other race category included persons of 2 or more races and those who self-reported race as Other. Race for patients who declined to answer or for whom race was not reported was considered “not reported.”

### Data collection

Trained infection prevention staff collected surveillance data using a standardized database and NHSN definitions. Device days and patient days were abstracted from an operational database linked to the electronic medical record. CLABSI and CAUTI rates were reported per 1,000 race- or ethnicity-specific catheter days.

### Analysis

We first performed descriptive analyses after stratification by racial and ethnic groups. CLABSI and CAUTI rates were then compared between the different racial and ethnic groups using Poisson regression. As a patient-centered measure of use and overall risk associated with indwelling catheters,^7^ catheter utilization ratios, calculated as the race- or ethnicity-specific catheter days divided by race- or ethnicity-specific patient days, were compared between the groups as a secondary outcome. Analyses were conducted in Stata release 17 software (StataCorp, College Station, TX).

## Results

During the 4-year surveillance period, 450 CLABSIs were reported during 439,224 catheter days (overall CLABSI rate, 1.02 per 1,000 catheter days). Race and ethnicity data were reported for 372 CLABSIs (82.7%) and 385,701 catheter days (0.96 per 1,000 catheter days). Similarly, 233 CAUTIs occurred during 217,590 urinary catheter days (overall CAUTI rate, 1.07 per 1,000 catheter days). Race and ethnicity data were reported for 193 CAUTIs (82.8%) and 188,984 catheter days (1.02 per 1,000 catheter days). Most CLABSIs and CAUTIs occurred in non-Hispanic White and non-Hispanic Black patients (Supplementary Tables 1 and 2 online).

CLABSI rates were significantly higher for non-Hispanic Black patients (1.27; 95% confidence interval [CI], 1.02–1.58; *P =* .03) and patients in the Other race category (2.25; 95% CI, 1.31–3.88; *P =* .003) compared with non-Hispanic White patients (Fig. 1a). Similarly, non-Hispanic Black (1.42, 95% CI, 1.05–1.92; *P =* .02) and Asian patients (2.49, 95% CI, 1.16–5.36; *P =* .02) had higher rates of CAUTI compared with non-Hispanic White patients (Fig. 1b).

**Fig. 1a.**
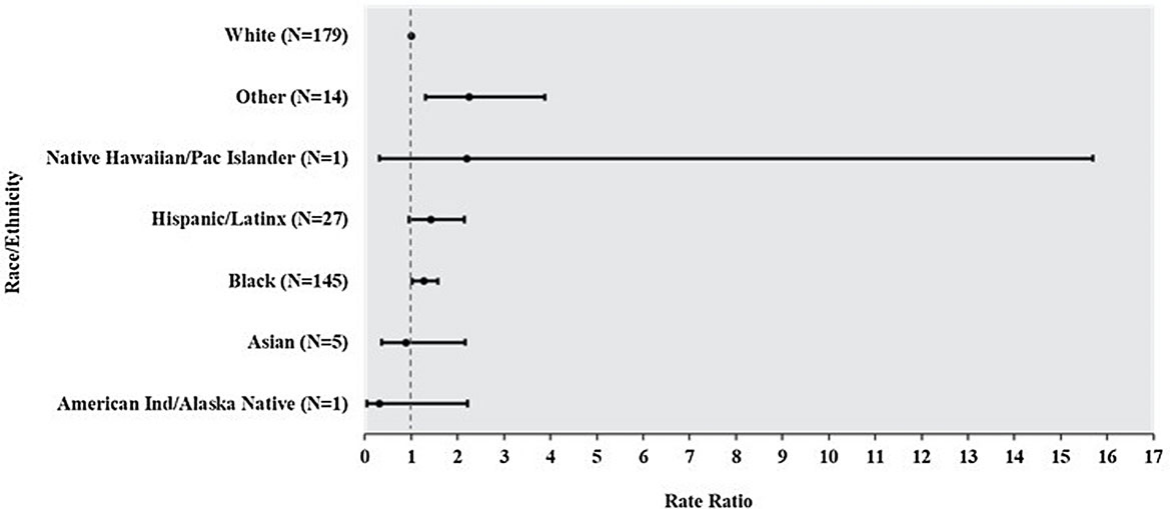
Total number of central line-associated bloodstream infections (CLABSIs) over the surveillance period by race and ethnicity and rate ratio of CLABSI by race and ethnicity. Non-Hispanic White was the reference group for all rate comparisons.

**Fig. 1b.**
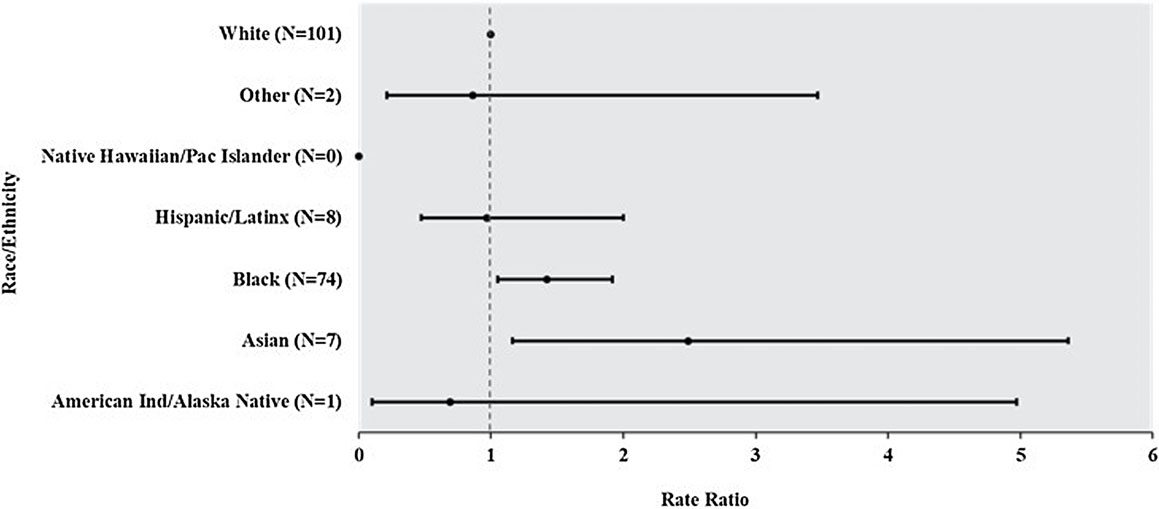
Total number of catheter-associated urinary tract infections (CAUTIs) over the surveillance period by race and ethnicity and rate ratio of CAUTI by race and ethnicity. Non-Hispanic White was the reference group for all rate comparisons.

Central line utilization rates among patients who identified as Black (0.30) or Other (0.31) were similar to White patients (0.31) (Fig. 2a). Urinary catheter utilization was lower in Black (0.13) and Asian (0.12) groups relative to White patients (0.17). The number and rates of each outcome did not differ significantly annually (data not shown).

**Fig. 2a.**
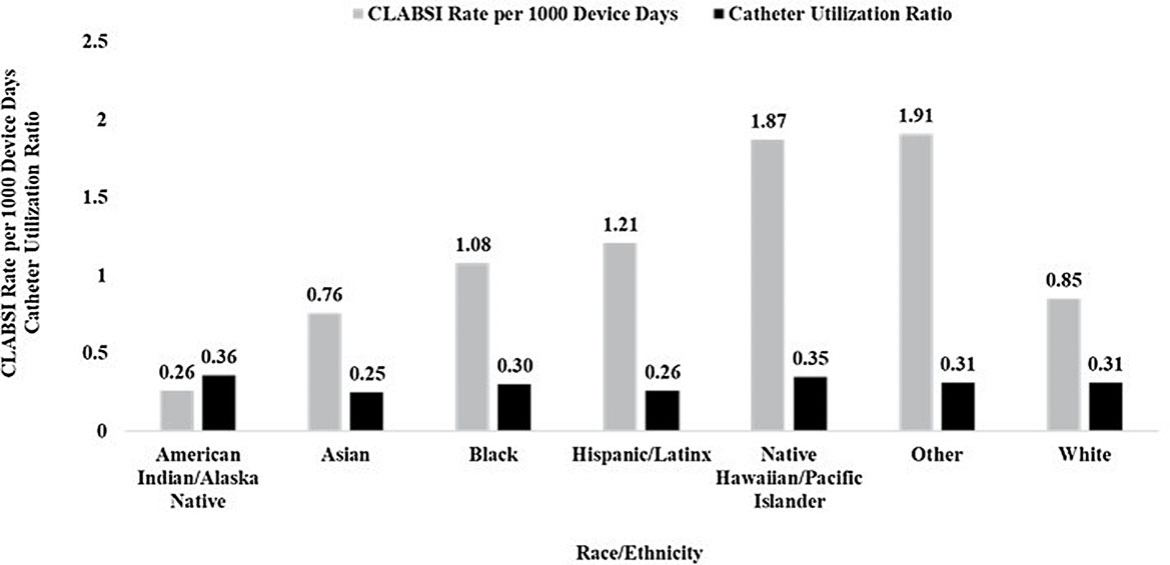
Rate of central line-associated bloodstream infection (CLABSI) and catheter utilization by race and ethnicity. Catheter utilization was calculated as race- or ethnicity-specific central line days divided by race- or ethnicity-specific patient days.

**Fig. 2b.**
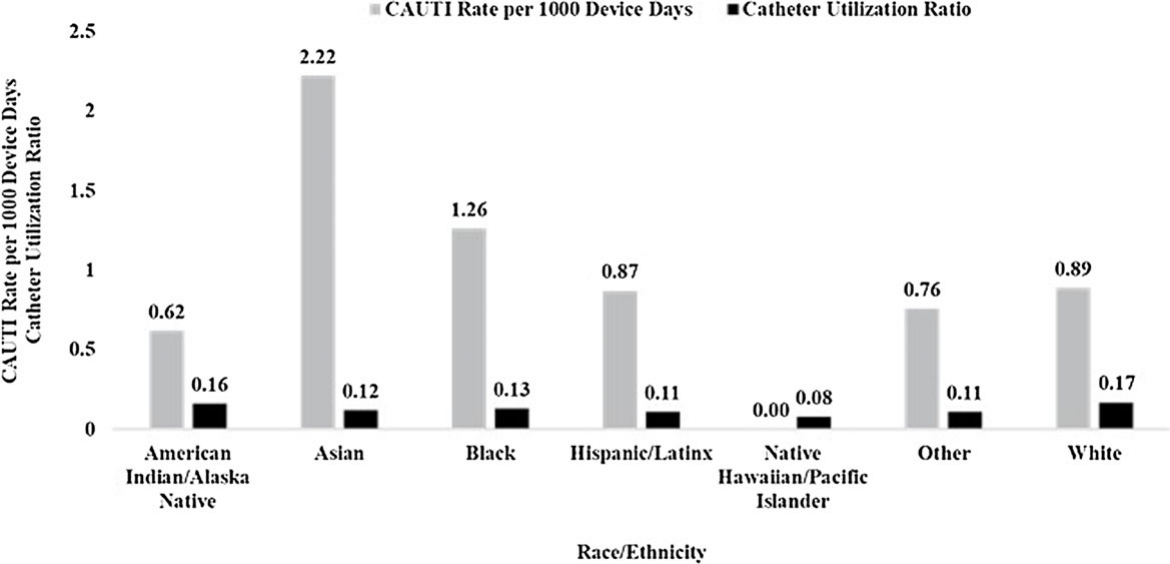
Rate of catheter-associated urinary tract infection (CAUTI) and catheter utilization by race and ethnicity. Catheter utilization was calculated as race- or ethnicity-specific urinary catheter days divided by race- or ethnicity-specific patient days.

## Discussion

This retrospective review of surveillance data revealed higher CLABSI rates in non-Hispanic Black patients and patients in the Other race category and higher CAUTI rates in Asian and non-Hispanic Black patients compared with non-Hispanic White patients. Despite similar catheter utilization among different racial and ethnic groups, we observed notable differences in CLABSI and CAUTI rates between documented racial and ethnic groups. Collectively, these observations may indicate potential HAI disparities that cannot be attributed to differences in catheter utilization or hospitalization rates.

Although race and ethnicity are social constructs with constantly evolving definitions, they remain significant markers of health and treatment outcomes. This correlation is the result of a complex, dynamic interplay across multiple socioecological levels, including unconscious bias among healthcare professionals, inequities in social and economic determinants of health, structural racism, chronic underfunding of safety net hospitals leading to healthcare segregation, insufficient research investigating the racial or ethnic inequities in HAI incidence, and other societal factors.^8^ Identifying and addressing contributors to healthcare inequities is critical. However, to do so accurately and effectively, we need improved documentation of self-reported race and ethnicity in medical records and surveillance databases. We detected a relatively high proportion of patients for whom race and ethnicity data were missing (17% for each outcome). A recent survey of 28 US hospitals also revealed gaps in collection of race, ethnicity, and social determinants of health.^9^ Our data also highlight the need to investigate inequities in clinical care, compliance with prevention toolkits, or disparities in process measures linked to HAIs (eg, blood culture utilization) across different racial and ethnic groups. For example, prior investigations have revealed that hospitalized Black patients were at higher risk of blood culture contamination.^10,11^

This study had several limitations. Race and ethnicity data were abstracted from the medical record and, as such, the accuracy of the documentation or concordance with patients’ self-reported data could not be confirmed. This was a single center, retrospective study with a relatively small number of observations, which may limit generalizability. Additionally, analyses were based on surveillance data at the individual HAI level rather than the patient level, and the outcomes were unadjusted for comorbidities or other potential confounding variables. Nevertheless, this study was intended to be exploratory and the results may be used to inform subsequent multivariate analyses.

In summary, minoritized populations in the United States may be vulnerable to higher HAI rates. These data provide evidence that supports expanding NHSN data fields to include race and ethnicity and recommending their input for key quality measures. Mandated reporting could allow for better understanding of existing inequities in HAI incidence across different sociodemographic groups. Reported data could subsequently inform investigations into drivers of increased HAI rates and identify modifiable targets for novel interventions in groups experiencing the greatest disparities. Importantly, this work has implications for policy changes on a national level to inform corrective strategies and provide optimum, equitable care for all.

## Supplementary material

For supplementary material accompanying this paper visit https://doi.org/10.1017/ice.2023.63. click here to view supplementary material
