# Supplementary material for: Disparities in central line-associated bloodstream infection and catheter-associated urinary tract infection rates: An exploratory analysis
Source: Infect Control Hosp Epidemiol. 2023 Apr 14;44(11):1857–60. doi: 10.1017/ice.2023.63 (PMC10665875; doi:10.1017/ice.2023.63)
Supplement: Supplementary file 1 [file S0899823X23000636sup001.docx]

**Supplemental Table 1. Rate and Rate Ratio (RR) of Central-Line Associated Bloodstream Infection (CLABSI) by Race and Ethnicity**

| **Race/Ethnicity** | **Total CLABSI**  **(n)** | **Rate^1^** | **RR** | **95% CI** | **p-value^2^** |
| --- | --- | --- | --- | --- | --- |
| American Indian/Alaska Native | 1 | 0.26 | 0.31 | 0.04-2.22 |  |
| Asian | 5 | 0.76 | 0.89 | 0.37-2.16 |  |
| Black | 145 | 1.08 | 1.27 | 1.02-1.58 |  |
| Hispanic/Latinx | 27 | 1.21 | 1.43 | 0.95-2.14 |  |
| Native Hawaiian/Pacific Islander | 1 | 1.87 | 2.20 | 0.31-15.70 |  |
| Other | 14 | 1.91 | 2.25 | 1.31-3.88 |  |
| White | 179 | 0.85 | Reference |  | 0.02 |
| Not reported/Missing | 78 | 1.46 |  |  |  |

^1^Rates of central line-associated bloodstream infection per 1000 catheter days.

^2^Overall p-value by chi-square listed with the reference group.

**Supplemental Table 2. Rate and Rate Ratio (RR) of Catheter-Associated Urinary Tract Infection (CAUTI) by Race and Ethnicity**

| **Race/Ethnicity** | **Total CAUTI**  **(n)** | **Rate^1^** | **RR** | **95% CI** | **p-value^2^** |
| --- | --- | --- | --- | --- | --- |
| American Indian/Alaska Native | 1 | 0.62 | 0.69 | 0.10-4.97 |  |
| Asian | 7 | 2.22 | 2.49 | 1.16-5.36 |  |
| Black | 74 | 1.26 | 1.42 | 1.05-1.92 |  |
| Hispanic/Latinx | 8 | 0.87 | 0.97 | 0.47-2.00 |  |
| Native Hawaiian/Pacific Islander | 0 | 0 | 0 | 0 |  |
| Other | 2 | 0.76 | 0.86 | 0.21-3.47 |  |
| White | 101 | 0.89 | Reference |  | 0.15 |
| Not reported/Missing | 40 | 1.40 |  |  |  |

^1^Rates of catheter-associated urinary tract infection per 1000 catheter days.

^2^Overall p-value by chi-square listed with the reference group.
